# Supplementary material for: Functional, Nutritional, and Sensory Quality of Mixed Flours-Based Breads as Compared to Durum Wheat Semolina-Based Breads
Source: Foods. 2021 Jul 12;10(7):1613. doi: 10.3390/foods10071613 (PMC8305921; doi:10.3390/foods10071613)
Supplement: Supplementary file 1 [file foods-10-01613-s001.zip › foods-1285489-supplementary.pdf]

Table S1. Gross chemical composition (in percentage of fresh matter) of the flours used in this study.

	Total carbohydrates	Sugars	Proteins	Total fats	Saturated fats	Fibres
Durum wheat semolina	73.0	0.4	13.0	2.0	0.4	3.6
Soft wheat (W=170)	73.0	0.4	10.0	2.0	0.4	2.0
Soft wheat (W=330)	69.0	1.5	13.0	0.7	0.2	2.2
Rye	65.0	1.5	12.0	1.8	0.4	8.0
Barley	78.0	0.8	10.3	1.8	0.4	7.1
Oat	62.0	1.2	12.6	7.1	1.3	7.6
Buckwheat	69.0	0.4	12.3	2.0	0.7	12.3

Table S2. Technology performances of mixed-flours sourdough (MFS) after 10 and 20 days of propagation. Values (mean of three independent experiments analysed in duplicate ± standard deviation) in the same row with at least one common superscript letter are not significantly different ($P > 0.05$).

	10 days	20 days
Cell density of lactic acid bacteria (log cfu/g)	9.3 ± 0.3^{a}	9.6 ± 0.3^{a}
Cell density of yeasts (log cfu/g)	7.4 ± 0.3^{a}	7.2 ± 0.2^{a}
Δ pH[§]	1.38 ± 0.05^{a}	1.36 ± 0.04^{a}
Δ V[†] (ml)	20 ± 1.0^{a}	20 ± 2.0^{a}

§ ΔpH was calculated as the difference between the values of pH at the beginning and end of daily fermentation; † Volume increase was calculated as the difference between the volume of the sourdough at the end and beginning of daily fermentation.

Table S3. Concentration (mg/kg) of volatile organic compounds detected in S-TMW, S-MFS, MF, MF-TWS, MF-MFS, and S breads.

Class of compounds	Compound	S-TWS	S-MFS	MF	MF-TWS	MF-MFS	S
Alcohols	ethanol	2.15±0.372ab	2.07±0.182b	0.96±0.100c	1.05±0.008c	0.98±0.007c	2.67±0.264a
	1-propanol, 2-methyl-	0.09±0.021b	0.08±0.007b	0.08±0.012b	0.06±0.001b	0.07±0.001b	0.13±0.007a
	1-butanol, 3-methyl	1.46±0.021b	1.24±0.012c	0.71±0.035e	0.82±0.033d	0.72±0.044e	1.95±0.017a
	1-hexanol	0.47±0.041a	0.23±0.008c	0.13±0.003d	0.23±0.011c	0.12±0.005d	0.28±0.003b
	2-furanmethanol	0.91±0.237a	0.61±0.006ab	0.69±0.073ab	0.57±0.101b	0.58±0.082b	0.83±0.003ab
	3-(methylthio)-1-propanol	0.87±0.236a	0.66±0.015ab	0.52±0.016b	0.51±0.049b	0.63±0.032ab	0.63±0.017ab
	3-nonen-1-ol,	0.25±0.035a	0.21±0.003a	0.09±0.009b	0.08±0.001b	0.08±0.003b	0.25±0.007a
	furfuryl alcohol	0.21±0.041a	0.21±0.011a	0.15±0.051a	0.13±0.067a	0.16±0.038a	0.18±0.013a
	benzyl alcohol	1.88±0.393a	1.06±0.011b	1.07±0.021b	1.44±0.032ab	1.17±0.042b	1.88±0.222a
	phenylethyl alcohol	16.53±2.718a	12.34±0.248b	5.89±0.778c	6.14±0.141c	6.62±0.269c	13.98±0.852ab
	maltol	0.32±0.053a	0.22±0.021ab	0.15±0.001b	0.15±0.019b	0.17±0.055b	0.24±0.033ab
	phenol	0.15±0.037a	0.11±0.014ab	0.14±0.036ab	0.11±0.033ab	0.07±0.008a	0.12±0.007ab
	2-butanol, 3-methyl-	0.20±0.026a	0.11±0.001c	0.06±0.01d	0.19±0.005ab	0.15±0.018b	0.00e
	2-methoxy-4-vinylphenol	0.14±0.067ab	0.15±0.011ab	0.12±0.024ab	0.12±0.001ab	0.2±0.034a	0.08±0.011a
	alpha-terpineol	0.00b	0.00b	0.08±0.046a	0.06±0.022a	0.08±0.004a	0.00b
Aldehydes	propanal, 2-methyl-	0.00b	0.01±0.014b	0.00b	0.00b	0.05±0.003a	0.00b
	butanal, 2-methyl-	0.00b	0.06±0.004a	0.06±0.009a	0.06±0.012a	0.06±0.004a	0.02±0.017b
	butanal, 3-methyl-	0.00c	0.14±0.008a	0.07±0.005b	0.11±0.008b	0.13±0.011a	0.02±0.018c
	hexanal	0.32±0.011a	0.29±0.008b	0.3±0.028ab	0.27±0.002b	0.28±0.006b	0.33±0.002a

	heptanal	0.06±0.056ab	0.00b	0.11±0.007a	0.09±0.018ab	0.05±0.045ab	0.04±0.042ab
	nonanal	0.23±0.088a	0.15±0.001a	0.21±0.008a	0.18±0.024a	0.17±0.003a	0.22±0.048a
	2-octenal	0.08±0.013abc	0.06±0.007c	0.07±0.021bc	0.11±0.007a	0.06±0.009c	0.11±0.004ab
	decanal	0.45±0.072a	0.34±0.002b	0.31±0.003b	0.28±0.046b	0.33±0.042b	0.35±0.008ab
	benzaldehyde	0.79±0.036ab	0.65±0.054bc	0.74±0.009abc	0.75±0.076abc	0.86±0.105a	0.58±0.027c
	2-nonenal	0.87±0.135a	0.64±0.011b	0.37±0.019c	0.37±0.084c	0.33±0.034c	0.69±0.064ab
	2-furancarboxaldehyde, 5-methyl	0.61±0.124b	0.78±0.012ab	0.69±0.062b	0.75±0.137ab	0.99±0.127a	0.35±0.015c
	2-phenyl-2-butenal	0.00b	0.06±0.003a	0.00b	0.00b	0.06±0.012a	0.00b
Alkanes	1-hexyl-2-nitrocyclohexane	0.04±0.038a	0.02±0.021a	0.06±0.002a	0.06±0.002a	0.06±0.001a	0.02±0.024a
	nonadecane	0.13±0.023b	0.12±0.006b	0.05±0.005c	0.05±0.001c	0.05±0.006c	0.18±0.002a
	pentadecane	0.18±0.053a	0.11±0.009b	0.00c	0.00c	0.00c	0.15±0.001ab
	heneicosane	0.08±0.011a	0.06±0.005a	0.08±0.001a	0.07±0.004a	0.07±0.012a	0.08±0.014a
	d-limonene	0.00c	0.00c	0.3±0.028b	0.26±0.005b	0.36±0.043a	0.00c
Benzene compounds	benzene, butyl-	0.00a	0.00a	0.05±0.053a	0.05±0.054a	0.07±0.068a	0.00a
	benzaldehyde, 2-hydroxy-	0.00b	0.00b	0.31±0.013a	0.27±0.057a	0.33±0.046a	0.00b
Esters	ethyl acetate	0.09±0.017ab	0.08±0.004b	0.09±0.007ab	0.08±0.005b	0.08±0.001b	0.11±0.009a
	hexanoic acid, 2-methyl-	0.14±0.031a	0.13±0.001a	0.00b	0.00b	0.05±0.049b	0.14±0.004a
	octanoic acid, ethyl ester	0.77±0.108ab	0.78±0.005ab	0.86±0.024ab	0.86±0.087ab	0.98±0.123a	0.69±0.019b
	2-octanoic acid, 2'-hexyl-,methyl ester	0.21±0.004a	0.15±0.004bc	0.12±0.008cd	0.11±0.019d	0.12±0.022cd	0.17±0.009b
	decanoic acid, ethyl ester	0.28±0.041a	0.22±0.003b	0.19±0.014bc	0.17±0.011bc	0.16±0.009c	0.31±0.004a

	benzeneacetic acid, ethyl ester	0.00c	0.00c	0.04±0.015b	0.05±0.008b	0.08±0.009a	0.00c
	acetic acid, 2-phenylethyl ester	0.26±0.048a	0.23±0.002a	0.09±0.009c	0.11±0.001c	0.11±0.005c	0.17±0.009b
	nonanoic acid, nonyl ester	0.29±0.055b	0.13±0.003c	0.25±0.007b	0.48±0.062a	0.14±0.001c	0.27±0.003b
	hexadecanoic acid, ethyl ester	0.11±0.021ab	0.07±0.004b	0.08±0.005b	0.1±0.011ab	0.07±0.018b	0.11±0.004a
Heterocyclic compounds	furan, 2-pentyl-	0.64±0.022a	0.37±0.026c	0.53±0.062ab	0.61±0.046a	0.58±0.083ab	0.46±0.012bc
	furfural	1.58±0.257bc	1.96±0.006b	2.35±0.163b	2.31±0.554b	3.17±0.311a	0.91±0.041c
	indole	0.15±0.059a	0.11±0.01ab	0.06±0.011b	0.07±0.016b	0.08±0.001ab	0.11±0.011ab
	benzeneacetaldehyde	0.32±0.039c	0.45±0.006b	0.35±0.001bc	0.38±0.048bc	0.78±0.072a	0.18±0.033d
	2-acetyl-pyridine	0.18±0.029ab	0.16±0.006b	0.29±0.016a	0.25±0.046ab	0.21±0.082ab	0.14±0.005b
	1h-pyrrole, 2-methyl-	0.07±0.017bc	0.07±0.003bc	0.11±0.006a	0.09±0.018ab	0.12±0.011a	0.05±0.006c
	1h-pyrrole, 1-(2-furanylmethyl)-	0.21±0.062bc	0.21±0.002bc	0.33±0.031b	0.31±0.079b	0.51±0.094a	0.12±0.024c
	2-acetyl-1-pyrroline	0.08±0.02ab	0.09±0.006ab	0.07±0.004ab	0.07±0.011ab	0.11±0.025a	0.06±0.002a
	1h-pyrrole-2-carboxaldehyde	0.00c	0.04±0.002b	0.05±0.011b	0.06±0.013b	0.11±0.031a	0.00c
	4-hydroxy-2,5-dimethyl-3(2h)-furanone	0.23±0.039a	0.16±0.007bc	0.09±0.003d	0.13±0.005cd	0.11±0.018cd	0.19±0.023ab
	1-furfuryl-pyrrol	0.00c	0.02±0.016bc	0.03±0.004bc	0.05±0.008b	0.11±0.027a	0.00c
Ketones	acetoin	0.16±0.022b	0.08±0.001c	0.24±0.041a	0.16±0.016b	0.07±0.006c	0.18±0.001b
	2-nonanone	0.32±0.046a	0.27±0.005abc	0.22±0.021bce	0.21±0.002d	0.21±0.004cd	0.27±0.018ab
Organic acids	acetic acid	0.61±0.167b	0.72±0.028b	0.29±0.003c	0.61±0.081b	1.11±0.065a	0.11±0.001c
	hexanoic acid	0.54±0.115a	0.42±0.006ab	0.26±0.024c	0.33±0.011bc	0.37±0.025bc	0.33±0.007bc
	octanoic acid	0.59±0.131ab	0.35±0.003b	0.31±0.129b	0.48±0.119ab	0.31±0.104b	0.73±0.091a

	n-hexadecanoic acid	0.08±0.075a	0.04±0.044a	0.00a	0.00a	0.00a	0.04±0.044a
	dodecanoic acid	0.05±0.051a	0.00a	0.00a	0.00a	0.00a	0.00a
Pyrazines	pyrazine, methyl-	0.64±0.167ab	0.58±0.017ab	0.73±0.057a	0.56±0.168ab	0.52±0.011ab	0.41±0.016b
	pyrazine, 2,5-dimethyl-	0.39±0.052a	0.42±0.016a	0.43±0.023a	0.37±0.053a	0.41±0.065a	0.35±0.021a
	pyrazine, 2,6-dimethyl-	0.35±0.047a	0.33±0.034ab	0.26±0.025ab	0.23±0.046b	0.24±0.053ab	0.32±0.021ab
	pyrazine, 2,3-dimethyl-	0.18±0.011ab	0.11±0.035bc	0.23±0.031a	0.18±0.047a	0.17±0.031ab	0.07±0.001c
	pyrazine, 2-ethyl-6-methyl-	0.41±0.05bc	0.44±0.006bc	0.69±0.088a	0.58±0.137ab	0.73±0.129a	0.28±0.003c
	pyrazine, 2-ethyl-5-methyl-	0.27±0.059a	0.28±0.002a	0.28±0.017a	0.25±0.081a	0.32±0.041a	0.11±0.004b
	pyrazine, 2-ethyl-3-methyl-	0.36±0.066ab	0.37±0.004ab	0.55±0.085a	0.47±0.123a	0.55±0.094a	0.23±0.003b
	pyrazine, 2,6-diethyl-	0.46±0.015a	0.46±0.039a	0.35±0.043ab	0.31±0.063b	0.41±0.073ab	0.38±0.001ab
	pyrazine, 3,5-diethyl-2-methyl	0.11±0.009b	0.14±0.002b	0.15±0.011b	0.15±0.042b	0.27±0.052a	0.08±0.006b
	pyrazine, (1-methylethenyl)-	0.11±0.016b	0.08±0.002b	0.11±0.003b	0.11±0.014ab	0.15±0.017a	0.03±0.029c
	pyrazine, 2-ethenyl-6-methyl-	0.11±0.005a	0.07±0.005cd	0.11±0.001ab	0.08±0.018bc	0.05±0.011d	0.12±0.009a
	pyrazine, ethyl-	0.29±0.07ab	0.26±0.007b	0.44±0.061a	0.37±0.073ab	0.45±0.102a	0.19±0.023b

Values (mean of three independent experiments analysed in duplicate ± standard deviation) in the same row with different superscript letters are significantly different (P < 0.05).
